# Combined HDL–BMI Phenotyping Provides Incremental Prognostic Value in Idiopathic Pulmonary Fibrosis

**DOI:** 10.3390/jcm15072525

**Published:** 2026-03-26

**Authors:** Qinxue Shen, Xiaoli Ouyang, Yuexin Tan, Qing Zhang, Feng Hu, Shengyang He, Hong Peng

**Affiliations:** 1 Department of Pulmonary and Critical Care Medicine, The Second Xiangya Hospital, Central South University, Changsha 410011, China; 2 Research Unit of Respiratory Disease, Central South University, Changsha 410011, China; 3 The Clinical Medical Research Center for Pulmonary and Critical Care Medicine in Hunan Province, Changsha 410011, China; 4 Diagnosis and Treatment Center of Respiratory Disease, Central South University, Changsha 410011, China; 5 Clinical Medical Research Center for Systemic Autoimmune Diseases in Hunan Province, Changsha 410011, China

**Keywords:** idiopathic pulmonary fibrosis, HDL–BMI, chronic disease prognosis, nutritional vulnerability

## Abstract

**Background/Objectives**: Risk stratification in idiopathic pulmonary fibrosis (IPF) remains primarily based on physiological indices, yet increasing evidence suggests that systemic metabolic and nutritional vulnerability may influence outcomes in chronic interstitial lung disease. **Methods**: In this longitudinal, single-center cohort, 211 patients with IPF were followed from diagnosis until death or last follow-up. Baseline lipid profiles and body mass index (BMI) were assessed. A metabolic–nutritional phenotype was constructed using high-density lipoprotein cholesterol (HDL) and BMI. Survival was analyzed using Kaplan–Meier and multivariable Cox models adjusted for GAP stage. Incremental prognostic value beyond the GAP index was evaluated using Harrell’s C-index and time-dependent ROC analysis. **Results:** During a median follow-up of 29 months, 134 patients (63.5%) died. Lower HDL levels were associated with increased mortality in unadjusted analysis (HR = 1.45, 95% CI 1.03–2.04) but were not independently predictive after adjustment. In contrast, the combined HDL–BMI phenotype independently stratified mortality risk. Compared with HDL ≤ 1.0 mmol/L and BMI ≤ 24 kg/m^2^, patients with HDL > 1.0 mmol/L and BMI > 24 kg/m^2^ had significantly lower mortality (adjusted HR = 0.48, 95% CI 0.29–0.80), with stronger associations among those aged ≥ 65 years (adjusted HR = 0.37, 95% CI 0.18–0.74). The addition of HDL–BMI improved discrimination beyond GAP (C-index: 0.585 vs. 0.618; 36-month AUC: 0.633 vs. 0.675; NRI: 0.243). **Conclusions**: The coexistence of HDL ≤ 1.0 mmol/L and BMI ≤ 24 kg/m^2^ identified a subgroup with poorer survival in IPF. This combined metabolic–nutritional phenotype improved mortality risk stratification beyond the GAP stage.

## 1. Introduction

Idiopathic pulmonary fibrosis (IPF) is a chronic, progressive interstitial lung disease characterized by irreversible fibrotic remodeling and poor long-term survival [1,2]. Despite advances in antifibrotic therapy, substantial heterogeneity in disease progression and outcomes persists, highlighting the need for improved risk stratification beyond conventional physiological and demographic parameters [1,3,4,5].

Increasing attention has been directed toward the role of nutritional status in the progression and prognosis of chronic respiratory diseases, as their clinical course is frequently accompanied by progressive weight loss, muscle wasting, and systemic metabolic alterations [6,7,8]. In IPF, even patients with normal body weight may exhibit underlying nutritional depletion, suggesting that body mass alone may not adequately reflect metabolic vulnerability [9]. These findings highlight the importance of assessing systemic metabolic and nutritional factors in addition to pulmonary function.

Emerging evidence indicates that IPF is associated with systemic metabolic disturbances and chronic inflammatory activation. Beyond pulmonary dysfunction, patients often present with abnormalities in lipid metabolism, energy homeostasis, and nutritional status [10,11,12]. Such alterations may reflect impaired physiological reserve and increased susceptibility to disease-related stress.

Lipid metabolism has emerged as an important component of IPF pathobiology. Experimental studies have demonstrated that dysregulated lipid processing contributes to epithelial injury and fibroblast activation [13,14]. Clinical and translational investigations have further identified altered circulating lipid profiles and distinct lipidomic signatures in IPF [15,16,17]. Several lipid-related biomarkers, including high-density lipoprotein (HDL)-associated parameters, have been reported to correlate with disease severity and survival [18]. However, circulating lipid concentrations are strongly influenced by systemic inflammation and metabolic status, and their independent prognostic value remains inconsistent.

Body composition and nutritional status are closely intertwined with lipid metabolism and systemic metabolic regulation [19]. Changes in body mass may reflect chronic inflammatory burden, altered lipid utilization, and energy imbalance in advanced lung disease [11,20,21]. Nevertheless, most previous studies have focused on individual lipid parameters in isolation, and the combined impact of metabolic and nutritional status on IPF prognosis has not been adequately explored.

Accordingly, we conducted a longitudinal cohort study to investigate the association between baseline lipid profiles and all-cause mortality in IPF. Building on exploratory analyses, we further examined the interaction between lipid parameters and nutritional status to determine whether an integrated metabolic–nutritional assessment could enhance risk stratification beyond established prognostic models in a real-world clinical setting.

## 2. Materials and Methods

### 2.1. Study Design and Population

This was a single-center, longitudinal cohort study using routinely collected clinical data and structured telephone follow-up. Consecutive patients diagnosed with IPF at the Second Xiangya Hospital of Central South University between January 2011 and December 2023 were included.

IPF was diagnosed according to the 2011 ATS/ERS/JRS/ALAT guidelines and confirmed by multidisciplinary discussion. Patients with severe respiratory failure or major organ dysfunction were excluded. Severe respiratory failure was defined as a resting PaO_2_ < 60 mmHg or the requirement for continuous oxygen therapy. Major organ dysfunction was defined as clinically significant cardiac, hepatic, or renal dysfunction requiring active treatment or hospitalization. Patients were followed from diagnosis until death, lung transplantation, loss to follow-up, or December 2023.

This study was approved by the Medical Ethics Committee of the Second Xiangya Hospital of Central South University (approval number LYF2022147, 22 September 2025). Oral informed consent to participate was obtained from all patients at the time of their initial clinical enrollment. All procedures were conducted in accordance with the Declaration of Helsinki.

### 2.2. Data Collection and Definitions

Baseline data at diagnosis included age, sex, smoking status, body mass index (BMI), high-resolution computed tomography pattern, and pulmonary function parameters. Forced vital capacity (FVC) and diffusing capacity for carbon monoxide (DLCO) were expressed as percentages of predicted values. The Gender–Age–Physiology (GAP) index and stage were calculated as previously described. Baseline fasting lipid profiles, including triglycerides, total cholesterol, HDL cholesterol (HDL-C), and LDL cholesterol (LDL-C), were obtained from routine laboratory testing at or near the time of diagnosis. All measurements were performed after an overnight fast. Patients were categorized into low- and high-HDL groups using a cut-off value of 1.0 mmol/L. Combined HDL–BMI phenotypes were defined using cut-off values of HDL (1.0 mmol/L) and BMI (24 kg/m^2^), resulting in four groups. Information on antifibrotic therapy and other treatments was collected from medical records.

### 2.3. Outcomes and Follow-Up

The primary outcome was all-cause mortality. Survival time was defined as the interval from diagnosis to death from any cause. Patients who were alive, lost to follow-up, or underwent lung transplantation were censored. Survival status was ascertained through telephone follow-up and medical record review.

### 2.4. Statistical Analysis

Continuous variables are presented as median (interquartile range), and categorical variables as number (percentage). Between-group comparisons were performed using the Mann–Whitney U test, χ^2^ test, or Fisher’s exact test, as appropriate.

Overall survival was estimated using the Kaplan–Meier method and compared using the log-rank test. Lung transplantation was treated as a censoring event in the survival analyses. Cox proportional hazards regression models were used to evaluate associations between lipid parameters and mortality, with adjustment for clinically relevant variables including GAP stage and BMI. Proportional hazard assumptions were assessed using Schoenfeld residuals. An interaction term between HDL and BMI was tested in multivariable Cox models to assess potential effect modification.

Model discrimination was evaluated using Harrell’s concordance index (C-index). Time-dependent receiver operating characteristic (ROC) curves were generated at 12 and 36 months to estimate the area under the curve (AUC). Incremental predictive value of the HDL–BMI phenotype beyond the GAP stage was assessed using net reclassification improvement (NRI). A sensitivity analysis excluding patients who died within 6 months of follow-up was performed.

Statistical analyses were conducted using SPSS version 25.0 (IBM Corp., Armonk, NY, USA), GraphPad Prism version 9.0, and R software (version 2025.09.2+418). A two-sided *p* value < 0.05 was considered statistically significant.

## 3. Results

### 3.1. Baseline Metabolic and Nutritional Characteristics

A total of 211 patients with IPF were included in the study. Baseline characteristics are summarized in Table 1. The median age was 65.0 years (IQR 58.0–71.0), with an age range of 41 to 90 years. More than half of the patients (54.0%) were aged ≥ 65 years. Most patients were male (79.6%) and had a history of smoking (67.8%).

The median BMI was 24.3 kg/m^2^ (IQR 22.1–26.7), with 52.6% having a BMI of ≥ 24 kg/m^2^. Pulmonary function indicated moderate disease severity, with a median FVC% predicted of 78.7% and DLCO% predicted of 52.0%. During a median follow-up of 29.0 months (IQR 17.0–44.0), 134 patients (63.5%) died.

Baseline lipid profiles are summarized in Table 2. Non-survivors had significantly lower HDL levels compared with survivors (1.00 vs. 1.07 mmol/L, *p* = 0.014), whereas triglycerides, total cholesterol, LDL cholesterol, and the HDL/total cholesterol ratio did not differ significantly between the groups.

### 3.2. Association Between HDL and Overall Survival

**Figure 1:**
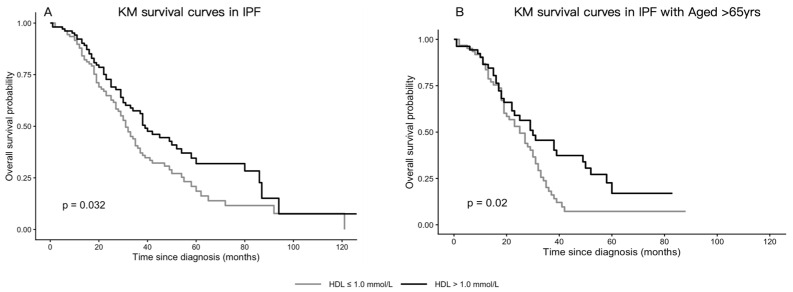
Kaplan–Meier survival curves by baseline HDL: (**A**) Overall cohort, stratified by HDL (≤1.0 vs. >1.0 mmol/L). (**B**) Patients aged ≥ 65 years, stratified by HDL.

In the overall cohort, patients with HDL ≤ 1.0 mmol/L (the cohort median) had significantly shorter survival than those with HDL > 1.0 mmol/L (log-rank *p* = 0.032), with median survival times of 31.0 and 39.0 months, respectively (Figure 1A). A similar separation of survival curves was observed among patients aged ≥ 65 years (Figure 1B). Restricted cubic spline analysis did not demonstrate a significant non-linear association between HDL and mortality (*p* for non-linearity = 0.578; overall *p* = 0.147) (Figure S1).

In univariate Cox regression analysis, HDL levels of ≤ 1.0 mmol/L were associated with an increased risk of mortality (HR = 1.45, 95% CI = 1.03–2.04, *p* = 0.034). After adjustment for the GAP stage, this association was attenuated and became borderline significant (adjusted HR = 1.40, 95% CI 0.99–1.98, *p* = 0.059). In the fully adjusted model including GAP stage, age, sex, and BMI, HDL ≤ 1.0 mmol/L was no longer independently associated with mortality (adjusted HR = 1.34, 95% CI 0.94–1.92, *p* = 0.107) (Table 3).

Although HDL showed prognostic relevance, its independent contribution appeared modest after adjustment for established clinical predictors. Given that circulating HDL levels are influenced by systemic inflammatory burden and nutritional reserve, we hypothesized that a combined metabolic–nutritional phenotype integrating HDL with BMI might better capture systemic vulnerability in IPF. To evaluate whether integrating metabolic and nutritional dimensions improves risk stratification, patients were categorized into four phenotypes according to baseline HDL (≤1.0 vs. >1.0 mmol/L) and BMI (≤24 vs. >24 kg/m^2^).

**Figure 2:**
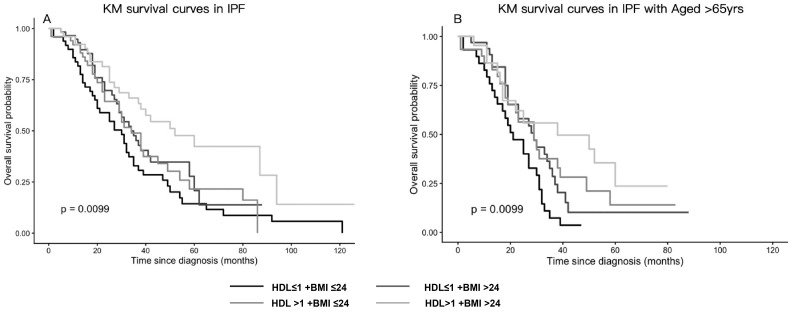
Kaplan–Meier survival curves by HDL–BMI phenotypes (HDL cut-off 1.0 mmol/L; BMI cut-off 24 kg/m^2^): (**A**) Overall cohort, stratified by HDL–BMI phenotypes. (**B**) Overall cohort, stratified by HDL–BMI phenotypes.

Kaplan–Meier analyses demonstrated significant survival differences among the four phenotypes in the overall cohort (log-rank *p* = 0.0099) and in patients aged ≥ 65 years (Figure 2). In both analyses, the HDL > 1.0 mmol/L + BMI > 24 kg/m^2^ group consistently exhibited the most favorable survival, whereas the HDL ≤ 1.0 mmol/L + BMI ≤ 24 kg/m^2^ group had the poorest outcomes.

No statistically significant interaction between HDL and BMI was observed (*p* for interaction = 0.900), suggesting additive rather than multiplicative effects.

### 3.3. Prognostic Impact of the Combined HDL–BMI Phenotype

**Figure 3:**
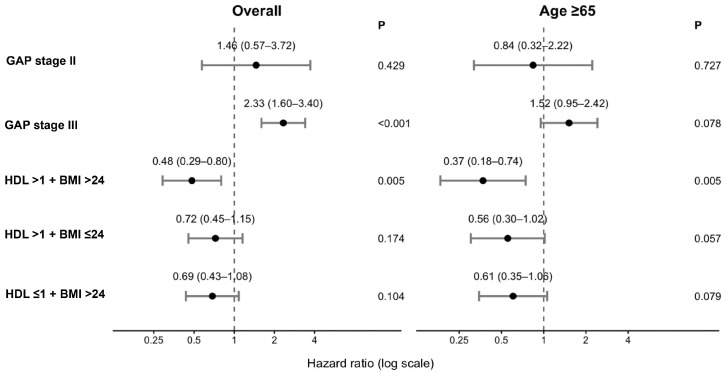
Multivariable Cox regression analysis of mortality risk (forest plot showing hazard ratios (HRs) and 95% confidence intervals (CIs) for HDL–BMI phenotypes and GAP stage in the multivariable Cox model). The reference group for HDL–BMI phenotypes was the group with HDL ≤ 1.0 mmol/L + BMI ≤ 24 kg/m^2^. GAP stage I was used as the reference category. The model was adjusted for the GAP stage. The vertical dashed line indicates HR = 1.0.

In Cox models adjusted for GAP stage, the HDL > 1.0 mmol/L + BMI > 24 kg/m^2^ phenotype was associated with significantly reduced mortality risk compared with the HDL ≤ 1.0 mmol/L + BMI ≤ 24 kg/m^2^ reference group in the overall cohort (HR = 0.48, 95% CI 0.29–0.80; *p* = 0.005) and in patients aged ≥ 65 years (HR = 0.37, 95% CI 0.18–0.74; *p* = 0.005). Multivariable hazard estimates are visualized in Figure 3. 

To assess the robustness of these findings, additional sensitivity analyses were performed with further adjustment for antifibrotic therapy and smoking status. The results remained materially unchanged across all models. In particular, the hazard ratio for the HDL > 1.0 mmol/L and BMI ≥ 24 kg/m^2^ group ranged from 0.48 to 0.50 after additional adjustments, indicating that the observed association was stable and not substantially influenced by these clinical factors (Supplementary Table S2).

These findings indicate that combined metabolic–nutritional phenotyping provides clearer prognostic separation than HDL alone.

### 3.4. Incremental Prognostic Value Beyond the GAP Index

We next evaluated whether the HDL–BMI phenotype improved risk stratification beyond the GAP index. The addition of the HDL–BMI phenotype resulted in a modest increase in discrimination, with Harrell’s C-index increasing from 0.585 for the GAP model to 0.618 for the combined model (ΔC = 0.033).

**Figure 4:**
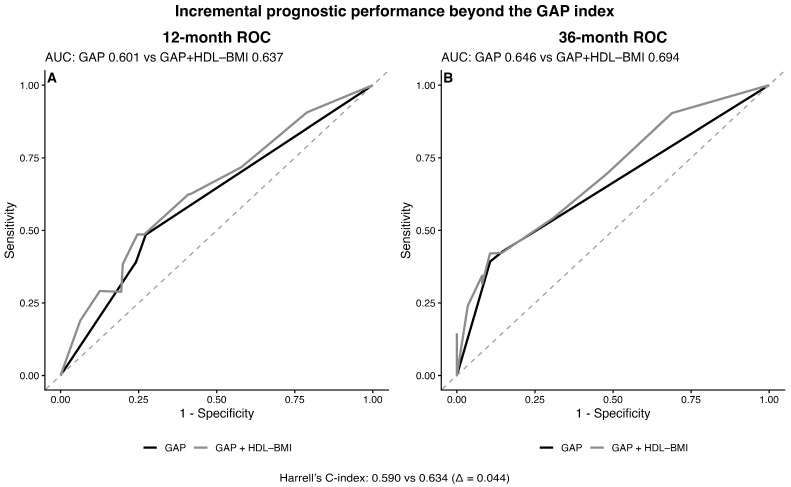
Incremental prognostic performance of the HDL–BMI phenotype beyond the GAP index. (**A**) Time-dependent ROC curves at 12 months comparing the GAP model and the GAP + HDL–BMI phenotype model. (**B**) Time-dependent ROC curves at 36 months comparing the GAP model and the GAP + HDL–BMI phenotype model. Addition of the HDL–BMI phenotype modestly improved discrimination. Harrell’s C-index increased from 0.585 to 0.618 (Δ = 0.033).

Time-dependent ROC analysis showed higher AUC values at both early and later time points (Figure 4). The AUC increased from 0.612 to 0.639 at 12 months and from 0.633 to 0.675 at 36 months. In addition, continuous net reclassification improvement (NRI) at 36 months was 0.243 (95% CI 0.057–0.419) (Figure S2), indicating improved long-term risk reclassification after incorporating the HDL–BMI phenotype.

### 3.5. Sensitivity Analysis

In sensitivity analyses excluding patients who died within the first 6 months of follow-up, the incremental prognostic improvement associated with HDL–BMI remained consistent (Supplementary Table S1), suggesting that the observed effects were not driven by early mortality.

## 4. Discussion

In this longitudinal cohort of patients with IPF, we found that baseline HDL levels were associated with survival, but their independent effect weakened after adjustment for disease severity. When HDL was considered together with BMI, the combined phenotype more clearly separated survival curves and remained independently associated with mortality. Importantly, this combined metabolic–nutritional phenotype remained independently associated with mortality and improved risk discrimination beyond the GAP stage. These findings indicate that metabolic vulnerability in IPF is better captured through integrated assessment rather than isolated lipid measurements.

Circulating HDL levels are strongly influenced by systemic inflammation, oxidative stress, and nutritional impairment, which may limit their independent prognostic value. Inflammatory responses can modify HDL composition and function, resulting in dysfunctional particles with impaired anti-inflammatory properties. In patients with chronic respiratory diseases, systemic inflammation is closely associated with disease severity and metabolic derangements [22]. In addition, recent observational analyses suggest that total cholesterol levels may also be associated with survival in IPF [23]. Taken together, these findings imply that serum HDL levels reflect broader systemic processes rather than functioning as independent determinants of prognosis. Thus, reduced HDL may represent a marker of systemic metabolic and inflammatory burden rather than a direct causal factor. This biological context may explain why HDL alone did not retain independent significance after multivariable adjustment in our cohort.

Beyond isolated lipid parameters, increasing experimental and translational evidence supports a broader role of metabolic dysregulation in pulmonary fibrosis. Altered lipid processing has been observed in fibrotic lung tissue, and metabolic profiling studies have identified distinct circulating lipid signatures in IPF. Moreover, specific lipid metabolites, such as 5-hydroxyeicosatetraenoic acid (5-HETE), have been proposed as potential biomarkers in acute exacerbations of IPF [24,25]. These findings indicate that lipid abnormalities reflect complex systemic dysfunction rather than simple changes in serum cholesterol levels.

Nutritional status represents another important determinant of disease resilience in IPF. The favorable prognostic impact of higher BMI observed in our study aligns with the “obesity paradox” reported in various chronic respiratory diseases and in IPF specifically [26,27]. Higher BMI may reflect greater energy reserves and resistance to catabolic stress, whereas low BMI often represents malnutrition, muscle wasting, and frailty. In our cohort, BMI ≥ 24 kg/m^2^ remained independently associated with reduced mortality risk, supporting the notion that preserved nutritional status plays an important role in disease resilience.

Importantly, the combined assessment of HDL and BMI integrates complementary aspects of metabolic health [28]. BMI reflects global nutritional and structural reserve [10], whereas HDL represents lipid transport capacity and systemic inflammatory balance [17,18]. The integration of these two dimensions may therefore better capture the interplay between metabolic dysregulation and nutritional depletion in IPF. Notably, similar HDL–BMI interaction patterns have been reported in other chronic diseases. For example, in a large prospective cohort study of stroke risk, the association between HDL and incident stroke was modified by BMI, suggesting that the prognostic relevance of HDL may depend on underlying anthropometric and metabolic status [21]. In cardiovascular and metabolic disorders, similar metabolic–anthropometric phenotyping approaches have been applied to improve risk stratification beyond conventional single markers [21,29,30]. Our findings extend this concept to IPF, suggesting that the interaction between lipid metabolism and nutritional status plays a critical role in shaping systemic vulnerability and clinical outcomes.

The combined HDL–BMI phenotype provided incremental prognostic information beyond the GAP index, which remains the most widely used clinical risk stratification tool in IPF. Although the GAP model incorporates key demographic and physiological variables, it does not capture metabolic or nutritional vulnerability [31].

The improvement in C-index and time-dependent AUC was modest but consistent and should be interpreted with caution. These findings suggest that the HDL–BMI phenotype is not intended to replace established prognostic models but may provide additional risk stratification by capturing aspects of systemic metabolic and nutritional status that are not fully reflected in physiological parameters. Given that HDL and BMI are routinely available clinical measures, even a modest improvement may have practical relevance in real-world settings.

The robustness of our findings was supported by sensitivity analyses excluding patients with early mortality, minimizing the likelihood that the observed associations were driven by reverse causation related to terminal disease status. This approach has been recommended to address bias in observational studies evaluating biomarkers in chronic diseases.

Several limitations should be acknowledged. First, this was a retrospective analysis conducted at a single tertiary referral center, which may limit the generalizability of our findings. Differences in population characteristics, nutritional status, and treatment practices across regions and healthcare systems may influence the applicability of these results to other clinical settings. Therefore, external validation in independent, multi-center cohorts is warranted. Second, only baseline lipid measurements were available, and changes over time were not assessed. Third, detailed assessments of body composition or HDL functionality were unavailable. Despite these limitations, the relatively large sample size, comprehensive clinical characterization, and consistent findings across analytical approaches strengthen the validity of our conclusions.

## 5. Conclusions

Lower HDL levels were associated with worse survival in IPF, but HDL alone did not independently predict outcomes after adjustment. A combined HDL–BMI phenotype provided better mortality stratification and modestly improved prediction beyond the GAP stage. Metabolic and nutritional assessment may therefore add clinically relevant information to traditional prognostic models.

## Figures and Tables

**Table 1:** Baseline characteristics of IPF patients.

Variable	Overall (n = 211)
Age (years)	65.0 (58.0–71.0)
Age ≥ 65 years (n, %)	114, 54%
Male (n, %)	168, 79.6%
Smoking (n, %)	143, 67.8%
BMI (kg/m^2^)	24.3 (22.1–26.7)
BMI ≥ 24 (n, %)	111 (52.6%)
Lung function test	
FVC (L)	2.25 (1.78–2.70)
FVC % predicted	78.7 (62.2–91.4)
FVC % predicted < 75% (n, %)	89 (42.2%)
FEV1% predicted	82.0 (67.0–94.0)
FEV1 (L)	1.87 (1.53–2.19)
FEV1/FVC (%)	90.0 (81.0–98.7)
DLCO % predicted	52.0 (35.1–67.0)
DLCO % predicted < 55% (n, %)	119 (56.4%)
Diagnosis time from symptoms (months)	24.0 (7.3–48.0)
Antifibrotic treatment	111 (53.4%)
Outcome	
Death (n, %)	134, 63.5%
Follow-up time (months)	29.0 (17.0–44.0)

Note: Continuous variables are presented as median (interquartile range), and categorical variables are presented as number (percentage). Differences between groups were compared using the Mann–Whitney U test for continuous variables and the χ^2^ test or Fisher’s exact test for categorical variables, as appropriate.

**Table 2:** Comparison of lipid profiles between survivors and non-survivors.

Variable	Overall (n = 211)	Survived (n = 77)	Non-Survivors (n = 134)	*p*-Value
Triglycerides (mmol/L)	1.36 (1.02–1.90)	1.31 (0.99–2.21)	1.37 (1.02–1.89)	0.749
Total cholesterol (mmol/L)	4.30 (3.72–4.91)	4.42 (3.85–5.07)	4.15 (3.58–4.87)	0.088
HDL (mmol/L)	1.00 (0.83–1.21)	1.07 (0.89–1.25)	0.96 (0.82–1.17)	0.014
LDL (mmol/L)	2.72 (2.18–3.26)	2.79 (2.31–3.28)	2.70 (2.11–3.27)	0.304
HDL/Total cholesterol	0.24 (0.20–0.28)	0.24 (0.20–0.29)	0.24 (0.20–0.28)	0.381
Triglycerides/HDL	1.36 (0.90–2.18)	1.32 (0.77–2.16)	1.42 (0.94–2.18)	0.173

**Table 3:** Multivariable Cox proportional hazards models for all-cause mortality.

Variable	Model 1: Unadjusted HR (95% CI)	*p* Value	Model 2	*p* Value	Model 3	*p* Value
HDL ≤ 1 mmol/L	1.45 (1.03–2.04)	0.034	1.40 (0.99–1.98)	0.059	1.342 (0.938–1.919)	0.107

Note: HR, hazard ratio; CI, confidence interval. Model 2 was adjusted by GAP stage; Model 3 was adjusted by GAP stage, age, sex, and BMI; HDL ≤ 1 mmol/L.

## Data Availability

The data presented in this study are available on request from the corresponding author.

## Supplementary Materials

The following supporting information can be downloaded at: https://www.mdpi.com/article/10.3390/jcm15072525/s1, Figure S1. Restricted cubic spline analysis of HDL and mortality risk.; Figure S2. Continuous net reclassification improvement (NRI) at 36 months after adding the HDL–BMI phenotype to the GAP model.; Table S1: Sensitivity Cox regression analysis after exclusion of early deaths. Table S2. Sensitivity analyses of HDL–BMI phenotype.

## Abbreviations

The following abbreviations are used in this manuscript:

IPF	Idiopathic pulmonary fibrosis
HDL	High-density lipoprotein cholesterol
LDL	Low-density lipoprotein cholesterol
BMI	Body mass index
GAP	Gender–Age–Physiology index
FVC	Forced vital capacity
DLCO	Diffusing capacity of the lung for carbon monoxide
HR	Hazard ratio
CI	Confidence interval
ROC	Receiver operating characteristic
AUC	Area under the curve
NRI	Net reclassification improvement
