# Supplementary material for: Combined HDL–BMI Phenotyping Provides Incremental Prognostic Value in Idiopathic Pulmonary Fibrosis
Source: J Clin Med. 2026 Mar 26;15(7):2525. doi: 10.3390/jcm15072525 (PMC13073493; doi:10.3390/jcm15072525)
Supplement: Supplementary file 1 [file jcm-15-02525-s001.zip › jcm-4206503-supplementary.pdf]

Table S1. Sensitivity Cox regression analysis after exclusion of early deaths

HDL–BMI phenotype	HR (95% CI)	P value
HDL ≤1.0 / BMI ≤24	1.00 (Reference)	—
HDL ≤1.0 / BMI >24	0.70 (0.44–1.11)	0.127
HDL >1.0 / BMI ≤24	0.72 (0.45–1.17)	0.184
HDL >1.0 / BMI >24	0.48 (0.29–0.80)	0.005
**GAP stage**		
GAP II vs I	2.25 (1.52-3.31)	<0.001
GAP III vs I	1.21 (0.43-3.40)	0.724

Note: HDL–BMI phenotypes were defined using median cut-offs (HDL 1.0 mmol/L; BMI 24 kg/m²). HRs were adjusted for GAP stage.

Table S2. Sensitivity analyses of HDL–BMI phenotype

Model	Adjustment	HDL ≤1.0 / BMI >24	HDL >1.0 / BMI ≤24	HDL >1.0 / BMI >24
Model 1	GAP stage	0.69 (0.43–1.08), P=0.104	0.72 (0.45–1.15), P=0.174	0.48 (0.29–0.80), P=0.005
Model 2	GAP + antifibrotic therapy	0.68 (0.43–1.07), P=0.092	0.73 (0.46–1.16), P=0.185	0.48 (0.29–0.80), P=0.004
Model 3	GAP + smoking	0.68 (0.43–1.07), P=0.097	0.77 (0.48–1.24), P=0.279	0.50 (0.30–0.84), P=0.008
Model 4	GAP + antifibrotic therapy + smoking	0.67 (0.43–1.06), P=0.089	0.77 (0.48–1.24), P=0.289	0.50 (0.30–0.84), P=0.009

Reference group: HDL ≤1.0 mmol/L and BMI <24 kg/m². Models were adjusted as indicated. HR: hazard ratio; CI: confidence interval.

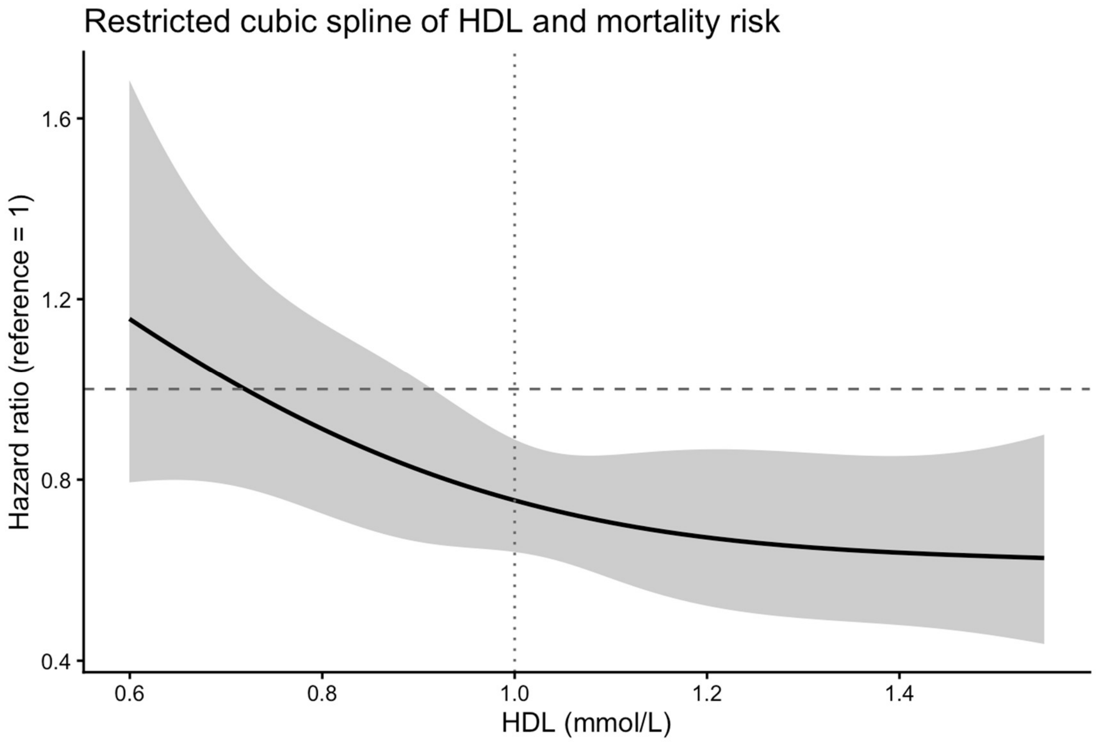

Figure S1. Restricted cubic spline analysis of HDL and mortality risk. Restricted cubic spline analysis adjusted for GAP stage showed no evidence of a non-linear association between HDL and mortality (P for non-linearity = 0.578). Shaded areas represent 95% confidence intervals. The vertical dotted line indicates the cohort median HDL value (1.0 mmol/L).

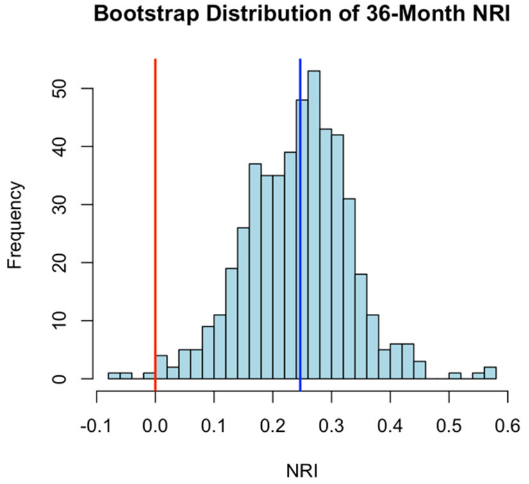

Figure S2. Continuous net reclassification improvement (NRI) at 36 months after adding the HDL–BMI phenotype to the GAP model. Continuous net reclassification improvement (NRI) was calculated to assess the incremental prognostic value of adding the HDL–BMI phenotype to the GAP model for 36-month mortality prediction. The addition of HDL–BMI

yielded a continuous NRI of 0.243 (95% CI 0.057–0.419), indicating improved long-term risk reclassification. Confidence intervals were derived from 500 bootstrap resamples.
